# Long‐term cardiac reverse remodeling after cardiac resynchronization therapy

**DOI:** 10.1002/joa3.12527

**Published:** 2021-03-21

**Authors:** Belén Alvarez‐Alvarez, Javier García‐Seara, Jose L. Martínez‐Sande, Moisés Rodríguez‐Mañero, Xesús A. Fernández López, Laila González‐Melchor, Diego Iglesias‐Alvarez, Francisco Gude, Carla Díaz‐Louzao, José R. González‐Juanatey

**Affiliations:** ^1^ Cardiology Department Clinical University Hospital of Santiago de Compostela Santiago de Compostela Spain; ^2^ Arrhythmia Unit Clinical University Hospital of Santiago de Compostela Santiago de Compostela Spain; ^3^ Epidemiology Department Clinical University Hospital of Santiago de Compostela Santiago de Compostela Spain; ^4^ Statistics and Biomedical Data Science Research Group (GRID‐BDS) Department of Statistics Mathematical Analysis and Optimization University of Santiago de Compostela Santiago de Compostela Spain

**Keywords:** cardiac resynchronization therapy, heart failure, long‐term follow‐up, ventricular remodeling

## Abstract

**Introduction:**

The benefit of cardiac resynchronization therapy (CRT) in heart failure (HF) patients with reduced left ventricular ejection fraction (LVEF) have been observed in the first year. However, there are few data on long‐term follow‐up and the effect of changes of LVEF on mortality. This study aimed to assess the LV remodeling after CRT implantation and the probable effect of changes in LVEF with repeated measures on mortality over time in a real‐world registry.

**Methods:**

Among our cohort of 328 consecutive CRT patients, mixed model effect analysis have been made to describe the temporal evolution of LVEF and LVESV changes over time up with several explanatory variables. Besides, the effect of LVEF along time on the probability of mortality was evaluated using joint modeling for longitudinal and survival data.

**Results:**

The study population included 328 patients (253 men; 70.2 ± 9.5 years) in 4.2 (2.9) years follow‐up. There was an increase in LVEF of 11% and a reduction in LVESV of 42 mL during the first year. These changes are more important during the first year, but slight changes remain during the follow‐up. The largest reduction in LVESV occurred in patients with left bundle branch block (LBBB) and the smallest reduction in patients with NYHA IV. The smallest increase in LVEF was an ischemic etiology, longer QRS, and LV electrode in a nonlateral vein. Besides, the results showed that the LVEF profiles taken during follow‐up after CRT were associated with changes in the risk of death.

**Conclusion:**

Reverse remodeling of the left ventricle is observed especially during the first year, but it seems to be maintained later after CRT implantation in a contemporary cohort of patients. Longitudinal measurements could give us additional information at predicting the individual mortality risk after adjusting by age and sex compared to a single LVEF measurement after CRT.

## Graphical abstract

It is a real‐world registry to evaluate the long‐term effect of CRT on LV reverse remodeling assessed by repeat LV structural and functional measurements. Left ventricular reverse remodeling is maintained after the first year of CRT implantation throughout the entire follow‐up in a real‐world registry. Repeat measurements of LVEF improve the risk of individual mortality after adjusting by age and sex during long‐term follow‐up compared with a only measurement of LVEF after CRT.

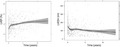

## 1 INTRODUCTION

Cardiac remodeling occurs due to abnormal neurohormonal regulation in heart failure (HF) patients.^1^, ^2^ The principal feature of remodeling is left ventricular (LV) dilatation and deterioration of LV function. Major clinical trials have demonstrated the benefit of cardiac resynchronization therapy (CRT) in HF patients with reduced LVEF.^3^, ^4^, ^5^, ^6^, ^7^, ^8^, ^9^ It is well established for patients with left ventricular systolic dysfunction and ventricular conduction delay under optimal medical therapy. CRT has been shown to improve functional class, reduce hospitalization and mortality among patients with symptomatic HF, as well as LV reverse remodeling. The concept of cardiac reverse remodeling was developed to explain the reduction of LV volumes and improvements in LV function observed with the use of medical therapies for HF and CRT.^10^ Currently, the reduction of left ventricular end‐systolic volume (LVESV) ≥15% and/or the increase in the absolute value of left ventricular ejection fraction (LVEF) ≥5% at 6‐12 months have been identified as predictors of clinical prognosis and response to CRT.^10^, ^11^, ^12^, ^13^ Evidence suggests that the improved outcomes observed with CRT are associated with reverse remodeling.^14^, ^15^, ^16^, ^17^

Up to date, randomized clinical trials of CRT have documented increases in LVEF and/or reduction in LV volumes in the first year after CRT implantation. However, there are scarce data on the mid‐term and long‐term benefit of this therapy and the sustained effect of ventricular remodeling over a prolonged follow‐up period. In addition, it remains unknown the effect of LVEF on mortality over time. Here, we evaluate the long‐term cardiac reverse remodeling after CRT in patients with repeated echocardiographic measurements in a contemporary patient registry and how changes in LVEF over time, with several measurements per patient, may better predict the individual risk of mortality.

## 2 METHODS

This retrospective follow‐up study includes 328 consecutive patients with cardiac resynchronization therapy—defibrillator (CRT‐D) or pacemaker (CRT‐P) under standard clinical indications in a single tertiary cardiac institution between January 2005 and April 2015. All the patients demonstrated HF symptoms (New York Heart Association—NYHA—functional class II, III or ambulatory IV symptoms), with ischemic or nonischemic cardiomyopathy, decreased LVEF (≤35%), and prolonged QRS duration (≥120 ms) at the time of implantation. They received pharmacological treatment for HF up‐titrated to the maximal tolerated doses according to the European Society of Cardiology guidelines for the management of HF at the discretion of the treating cardiologist.

We registered the baseline characteristics of all of the patients. Electrocardiographic features included QRS width and morphology. Echocardiographic parameters included LV end‐diastolic (LVEDV) and end‐systolic volume (LVESV), LVEF, left atrial diameter (LAD), and mitral regurgitation. For the quantification of the severity of mitral regurgitation, the ratio between the maximum regurgitant jet obtained from the flow image by color doppler and the area of the left atrium was used. In cases of eccentric mitral regurgitation, the contracted vein was used for quantification. The patients were followed up in the Heart Failure Clinic every 3 or 6 months and in the CRT‐Device Clinic every 6 months. Electrocardiogram and echocardiogram were also performed at the 6‐month and 2‐year follow‐ups and according to the discretion of the HF cardiologist. Treating cardiologists followed a specified protocol to achieve OMT. Patients with decreases in LVESV exceeding 15% and/or improvements in LVEF of more than 5% were considered to be echocardiographic responders. Patients with improvements in one category in NYHA functional class were considered to be clinical responders.

This study was conducted in accordance with the Principles of the Declaration of Helsinki. The clinical study (2017/171) was initially approved in 2017 by the Clinical Research Ethics Committee (CEIC) of Galicia.

### 2.1 Statistical analysis

Qualitative variables are expressed as frequency and percentages. Quantitative variables are presented as mean (SD) or as a median (interquartile range) as appropriate. The chi‐square test X2 or U of Mann‐Whitney was used to compare qualitative variables. *P* ≤ .05 was considered statistical significance.

Mixed effects models have been fitted to describe the relationship between echocardiographic changes (LVEF and LVESV) in the follow‐up as a function of several explanatory covariates (age, sex, etiology, NYHA class, rhythm in the time of implantation, morphology, and duration of the baseline QRS, location of the electrode in the coronary sinus). These models contain both fixed and random effects, possibility of correlated data, and variability heterogeneous.^18^ They are particularly useful in settings where repeated measurements are made on the same units (longitudinal study). Besides, this model allows to deal with missing values.

Joint modeling for longitudinal and survival data was used to model the relationship between LVEF profiles over time and mortality. These models allow dynamic predictions of the risk of mortality of individuals updating and improving with every new longitudinal observation.^19^ All statistical analyses were performed with the software R (CRAN‐R).^20^

## 3 RESULTS

### 3.1 Patient characteristics

The study population included 328 patients (253 men and 75 women; mean age: 70.2 (9.5) years) who were consecutively implanted with a CRT device at our institution. The mean follow‐up duration was 4.2 (2.9) years. Of the 328 patients, only 122 (37.2%) were on triple neurohormonal therapy at the time of implantation. The baseline characteristics are listed in Table 1.

**TABLE 1:** Baseline characteristics

Characteristics	n = 328
Sex: female, n (%)	75 (22.9)
Age (y)	70.2 (9.5)
Ischemic cardiomyopathy, n (%)	119 (36.3)
CRT‐D, n (%)	211 (64.3)
NYHA class, n (%)	
II	79 (24.1)
III	230 (70.1)
IV	19 (5.8)
Diabetes Mellitus, n (%)	77 (23.5)
Atrial fibrillation, n (%)	123 (37.5)
AV node ablation, n (%)	23 (7.0)
Creatinine (mg/dl)	1.3 (0.6)
Hemoglobine level (g/dl)	13 (2.0)
Coronary sinus vein, n (%)	
Anterior	74 (22.6)
Lateral	170 (51.8)
Posterior	84 (25.6)
QRS duration (ms)	162 (26.0)
QRS morphology, n (%)	
LBBB	198 (60.4)
RBBB	23 (7.0)
IVCD	39 (11.9)
RVP	68 (20.7)
Pharmacotherapy	
BB, n (%)	271 (82.6)
ACEI/ARB‐II, n (%)	283 (86.3)
MRA, n (%)	154 (47.0)
Echocardiographic parameters	
LVESV (mL)	215 (64)
LVEDV (mL)	156 (55)
LVEF (%)	28 (8)
Mitral regurgitation	
0	13 (4.0)
I	48 (14.6)
II	85 (25.9)
III	182 (55.5)
Left atrial volume (mL)	72 (15)

Abbreviations: ACEI, angiotensin‐converting enzyme inhibitors; ARB‐II, angiotensin II receptor blockers; AV, atrio ventricular; BB, beta‐blockers; CRT‐D, cardiac resynchronization therapy ‐ defibrillator; IVCD, intraventricular conduction delay; LBBB, left bundle branch block; LVEDV, left ventricular end‐diastolic volume; LVEF, left ventricular ejection fraction; LVESV, left ventricular end‐systolic volume; MRA, mineralocorticoid receptor antagonist; NYHA, New York Heart Association; RBBB, right bundle branch block; RVP, right ventricular pacing.

### 3.2 Baseline echocardiographic characteristics

The mean baseline LVEF was 28%. In our sample more than half of the patients had severe mitral regurgitation at the time of the CRT implant due to severe dilatation of the LV (182, 55.5%) and 13 (4.0%) did not present mitral regurgitation prior to CRT. LVESV and LVEDV were 156 (55) mL and 215 (64) mL, respectively (Table 1).

### 3.3 Mortality at follow‐up

The probability of survival at 1 year was 89.2%, at 5 years was 61.3% and at 10 years was 33.4%. Six patients were transplanted at 3 years after undergoing the CRT. During the first year of follow‐up, 34 (10.4%) patients died; 55.9% were cardiovascular mortality, mostly due to heart failure (73.7%). The causes of mortality were: 44.0% (55) cardiovascular mortality (74.5% HF, 21.9% arrhythmias, 3.6% coronary syndromes), 30.4% (38) no cardiovascular mortality (60.5% infections, 21.1% neoplasia, 18.4% bleeding events), and 25.6% (32) other and unknown.

### 3.4 Echocardiographic characteristics at follow‐up

The echocardiogram was performed during the first year and then every 2 years, at the request of the responsible clinician or during the patient's clinical deterioration. At follow‐up, a maximum of four measurements of the echocardiographic parameters were performed in patients with CRT. The first postimplant echocardiogram was obtained in 327 patients (one patient died during implant admission), the measurements of two echocardiograms were obtained in 181 patients, three measurements in 51 patients, and four measurements in 10 patients.

**FIGURE 1:**
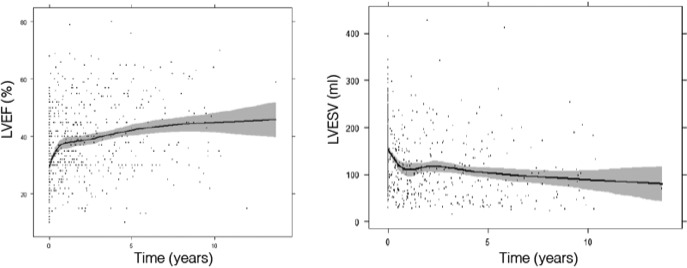
Evolution of changes in left ventricular ejection fraction (LVEF) and left ventricular end‐systolic volume (LVESV) during follow‐up

In the first year, LVEF increased by 11% and LVESV decreased by 42 mL. We have also identified that 68 patients (20.7%) presented normalization of the function and structure of the LV, associated with a better clinical prognosis. The changes in LVEF and LVESV profiles are observed especially during the first year, but improvements in LVEF and LVESV are not only maintained, but slightly increase in a longer follow‐up (Figure 1). This may be influenced because patients who die are excluded, 10.4% died during the first year and 38.2% at the end of follow‐up. Of them, 12 (3.7%) patients had sudden death.

Changes are observed during the first year in both sexes, but more marked in women than in men. It is also observed in patients with nonischemic cardiomyopathy. However, the response to CRT in patients with ischemic HF is unchanged in the long term. Patients in NYHA III class, and with LV lead located in posterior and lateral veins have better reverse remodeling of LV (Figures [Link], [Link], [Link], [Link], [Link]).

**FIGURE 2:**
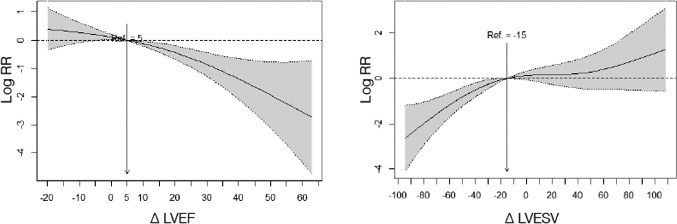
Effects of LVEF and LVESV changes on mortality risk. Interpretation: Taking the value of 5 on the X axis as the reference value for the change in LVEF, the logarithm of the relative risk of mortality for the change in LVEF is shown. For example, a 30‐point improvement in LVEF decreases the log RR by −1, which means that the relative risk of death is 2.72 times lower than a 5‐point improvement in LVEF. For the LVESV value, the reference value is 15. For LVESV reductions of 30, they will present a reduction in the relative risk of mortality 2.72 times greater than changes in LVESV of 15 (the number e, used as the base for a logarithm, is approximately equal to 2.72)

**FIGURE 3:**
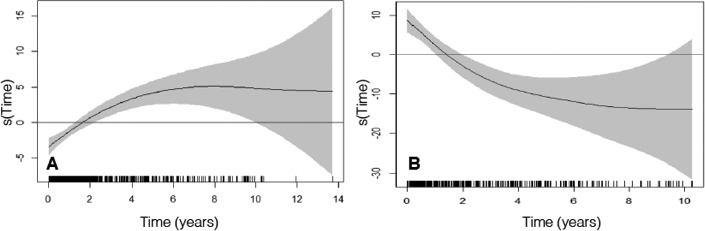
Evolution of the change of LVEF (A) and LVESV (B) adjusted for confounding variables over time (LVEF: *P* < .001 with 2.42 degrees of freedom; LVESV: *P* < .001 with 2.45 degrees of freedom)

The increases in LVEF greater than 5% and reductions in LVESV greater than 15% were associated with a reduction in mortality risk (Figure 2). Table 2 shows the variables associated with changes in LVEF and LVESV during follow‐up. The largest reduction in LVESD occurred in patients with LBBB (left bundle branch block), and the smallest reduction in patients with advanced functional class (NYHA IV class). The smallest increase in LVEF was observed in patients with ischemic etiology, longer QRS and location of the VI lead in a nonlateral vein of coronary sinus. Figure 3 shows the changes of the LVEF and LVESV along time after adjusting by potential confounding variables.

**TABLE 2:** Multivariable analysis (mixed model effects) to predict the change in LVEF (*P* < .0001 with degrees of freedom (*df*) 2.42) and LVESV (*P* < .001 with degrees of freedom (*df*) 2.45) over time

Variable	Coefficient	Standard error	*P*‐value
*Mixed model LVEF*			
Male	−2.096	1.64	.2019
Age (y)	0.013	0.07	.8495
Ischemic cardiomyopathy	−3.09	1.398	.0275
NYHA class			
III	−2.137	1.529	.1628
IV	−0.826	3.018	.7843
AF	−1.095	1.365	.4228
LBBB	1.367	1.341	.3084
QRS duration	−0.068	0.025	.0069
Coronary sinus vein			
Lateral	3.458	1.715	.0443
Posterior	3.514	1.969	.0648
Intercept	22.907	6.328	.0003
*Mixed model LVESV*			
Male	6.119	4.766	.1999
Age	−0.064	0.193	.7409
Ischemic cardiomyopathy	7.005	4.053	.0848
NYHA			
III	6.839	4.016	.0894
IV	31.88	10.463	.0025
AF	2.397	3.833	.5321
LBBB	−7.923	3.809	.0382
QRS duration	0.075	0.076	.3222
Coronary sinus vein			
Lateral	−6.481	5.549	.2435
Posterior	−10.419	6.052	.086
Intercept	−37.678	18.029	.0373

Abbreviations: AF, atrial fibrillation; LBBB, left bundle branch block; NYHA, New York Heart Association.

### 3.5 Joint modeling analysis

A survival prediction model has been carried out over time adjusted on age, sex, and changes of LVEF over time using the joint modeling methodology. Table 3 shows the relative risks with their corresponding confidence intervals. The analysis showed that the increase in LVEF after CRT was associated with higher survival rates. Male sex and older age were associated with a worse prognosis.

**TABLE 3:** Multivariable Analysis (Joint model) for mortality in HF patients after TRC during long‐term follow‐up

	Coefficient (SE)	HR (CI 95%)
Age (y)	0.05 (0.01)	1.05 (1.03‐1.07)
Male	0.57 (0.23)	1.57 (1.12‐2.77)
LVEF, % (∆)	−0.03 (0.01)	0.97 (0.95‐0.98)

Abbreviations: CI, confidence interval; HR, hazard ratio; LVEF, left ventricular ejection fraction; SE, standard error; ∆, changes.

## 4 DISCUSSION

This study provided a real‐world registry to evaluate the long‐term effect of CRT on LV reverse remodeling assessed by longitudinal LV structural and functional measurements over time in HF patients. CRT promotes a greater degree of reverse remodeling during the first year postimplant that is slightly enhanced in the long‐term follow‐up, especially in patients of female and nonischemic etiology. Moreover, variations of LV remodeling over time, analyzed using mixed models, have allowed us to improve the identification of reverse remodeling predictors. The main factors independent for predicting the improvement of LVEF have been the nonischemic etiology, higher duration of baseline QRS, and location of the LV lead in the lateral vein of the coronary sinus. The main factor for the prediction of the reduction of LVESV has been the presence of LBBB. Finally, the longitudinal changes in LVEF at follow‐up improves the prediction of the mortality risk after adjusting for age and sex, by using the joint modeling analysis. To our knowledge, this is the first time to describe the effect of CRT on LV remodeling in a real‐world registry. Our results have several implications, on the one hand, we describe the temporal evolution of cardiac morphology and function after CRT in a contemporary cohort of patients at follow‐up. On the other hand, we showed that the effect of CRT is observed in the long term, but especially in the first year. So it would be essential to optimize pharmacological treatment during this period in order to obtain the greatest benefit early, especially in patients with a worse response profile to CRT.

Reverse remodeling is one of the hallmark findings of CRT studies in mild and advanced HF patients. So far, there are few data on the mid‐ and long‐term of the ventricular remodeling.^10^, ^11^, ^12^, ^13^, ^14^, ^15^, ^16^, ^17^ It was well established the effect in the first 6‐12 months after CRT. This was assessed in randomized clinical trials, that showed that CRT added to standard pharmacological therapy for HF, causes a sustained improvement in LV geometry and function in the first months after CRT. In patients with HF, pharmacological agents and CRT reduce the mortality and morbidity, also improve the geometry and function of the LV. Reverse ventricular remodeling was first evaluated in the PATH‐CHF study at 12 months later to the implant and from there multiple studies have assessed the echocardiographic response to CRT and determined what characteristics could influence the reverse remodeling.^21^

Nowadays, the assessment of left ventricular remodeling after CRT is determined with the reduction of LVESV ≥ 15% or the increase in the absolute value of LVEF ≥ 5% at 6‐12 months. It is well established that the LV remodeling induced by CRT has been related to the improvement of the quality of life, functional class, reduction of mortality, and HF hospitalizations.^22^, ^23^ Most clinical trials have evaluated the LV remodeling during the 6‐12 months after CRT implantation. But so far, there is little evidence of the effect of CRT on LV remodeling during longer term follow‐up in real‐world. This study describes for the first time in a real‐world contemporary registry the long‐term effect of CRT on LV remodeling.

In a sub‐analysis of CARE HF trial, CRT induced sustained LV reverse remodeling in the long‐term follow‐up with the most marked effects occurring within the first 3‐9 months. This effect may contribute to an improvement in morbidity and mortality. These beneficial effects were observed even in ischaemic patients and patients with very severe cardiac dysfunction.^24^ MIRACLE study investigated serial changes in LV size and function by using longitudinal data analysis in a consecutive cohort of patients with long‐term follow‐up. These changes were more pronounced at 6 months and remained during the long‐term, but they became much less pronounced. Besides, patients with an uneventful survival demonstrated a greater decrease in the LVESV compared with patients with adverse events. Factors associated with less reverse remodeling were ischemic etiology, male sex, and QRS duration <140 ms.^25^ Similarly, the reverse LV remodeling of CRT in a sub‐analysis of REVERSE trial was sustained over 5 years. The functional and LV remodeling improvements were maximal after 2 years and were accompanied by very low mortality and HF hospitalization, although the largest change in LV volumes was noted in the first year with further remodeling.^26^

We show for the first time in a registry of real‐world patients the variation of echocardiographic measures over time and factors involved in their variations long‐term follow‐up. One of the main findings is the maintaining of a favorable evolutionary profile over years after the first year when the most LV reverse remodeling was observed. We observed better ventricular remodeling in women, nonischemic etiology, LBBB morphology, and LV lead in a lateral vein of the coronary sinus similar with randomized CRT trials. We have also observed that the evolutionary patterns of LVEF and LVESV have been very heterogeneous, due to both the individual characteristics of each patient, the location and type of lead in the coronary sinus and intercurrent processes that they could appear in the natural history of HF. These could be explained because more than a quarter of patients will not present an adequate echocardiographic response, despite good patient selection.^27^ However, Burns et al showed that the time course of improvements in LV size and function after the initiation of CRT can evidence a slower and delayed positive CRT response after 6‐12 months of therapy.^28^ These findings could also explain the maintaining of the long‐term ventricular remodeling that we have observed in our work. Furthermore, after CRT implantation there could be a hemodynamic improvement and thus a greater prescription of well evidence‐based treatments. Then, the synergistic effect of CRT and pharmacological treatment could improve structural remodeling during follow‐up. Besides, we have established, through dynamic prediction models, the individualized probability of patient survival through changes in LVEF with repeat measurements over time. This provides more information on mortality risk than a single determination of LVEF after implantation of CRT, after adjusting by age and sex. In our sample, the survival individually prediction through the models of dynamic prediction with repeated measurements of LVEF in follow‐up, could help to identify patients at higher risk who require closer monitoring, optimization of drug treatment and / or management of comorbidities. These models are probably the future of personalized medicine and could allow to establish computational algorithms of response to CRT that allow prediction what is the individual risk of both mortality and cardiovascular events at a specific time during follow‐up of each patient.

## 5 LIMITATIONS

The retrospective nature of this analysis conducted at a single center is a potential weakness. As a consequence, the patient sample size was limited. This is an observational registry with their inherent limitations (eg selection bias, unmeasured bias), and thus associations may be confounded by unmeasured variables. Several unmeasured confounders or details about physician or patient decision‐making might not be available in our collection data protocol and could account for some of the reported findings. Also, there may have been appropriate contraindications to adjunctive pharmacotherapy that were nor collected. In addition, during the echocardiographic follow‐up, patients who died are excluded from the analysis and this can be a positive bias because only surviving patients were included in the analysis. Finally, long‐term outcomes could be modified by many circumstances that might not be available or controlled in the follow‐up protocol of our center. As such, the results presented in this analysis should be considered hypothesis‐generating and deserve confirmation in other registries and clinical trials.

## 6 CONCLUSIONS

Reverse remodeling of the left ventricle is observed especially in the first year after CRT implantation and this effect is maintained a long time in a contemporary cohort of patients. Longitudinal LVEF measurements throughout the follow‐up could predict better the individual mortality risk adjusting for potential confounding variables.

## CONFLICT OF INTEREST

The authors declare no conflict of interests for this article.

## Supporting information

Fig S1

Fig S2

Fig S3

Fig S4

Fig S5
