# Supplementary figures and images for: Long‐term cardiac reverse remodeling after cardiac resynchronization therapy
Source: J Arrhythm. 2021 Mar 21;37(3):653–9. doi: 10.1002/joa3.12527 (PMC8207409; doi:10.1002/joa3.12527)

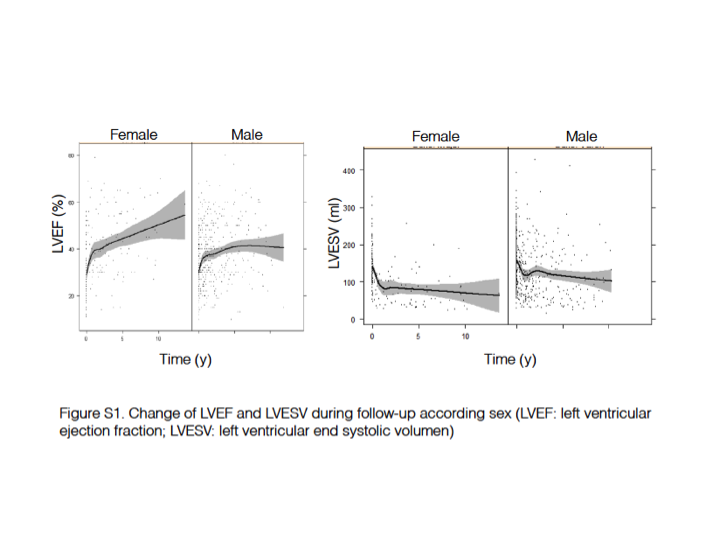

Supplement: Supplementary file 1 — Fig S1 [file JOA3-37-653-s003.tiff]

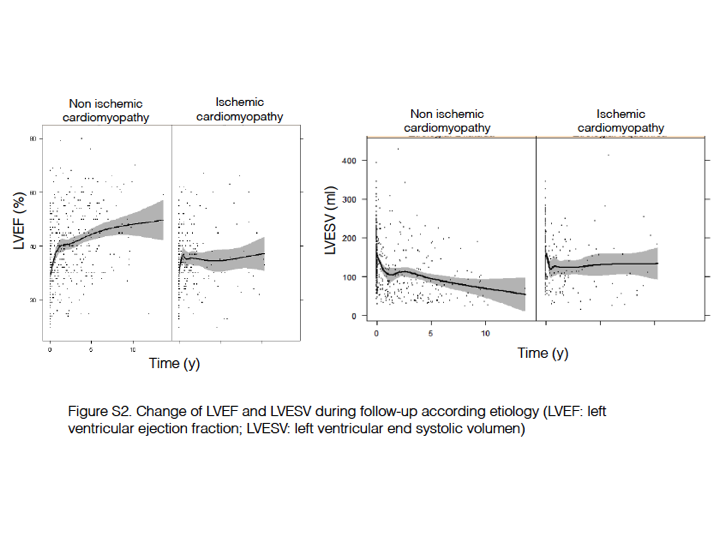

Supplement: Supplementary file 2 — Fig S2 [file JOA3-37-653-s004.tiff]

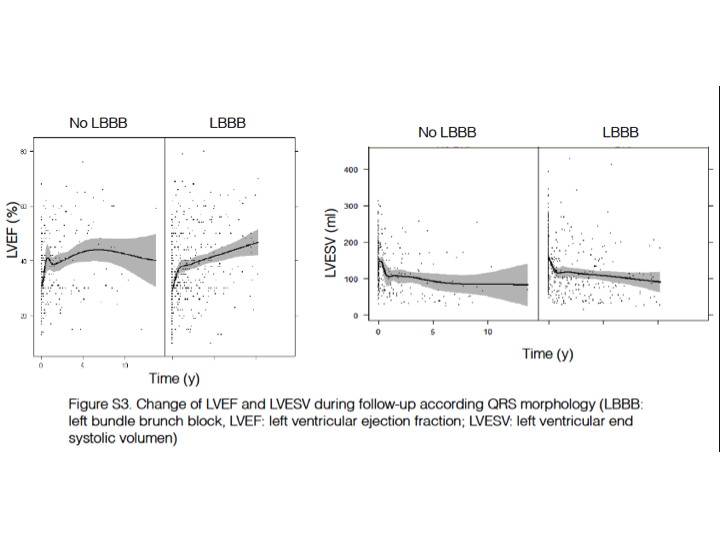

Supplement: Supplementary file 3 — Fig S3 [file JOA3-37-653-s001.tiff]

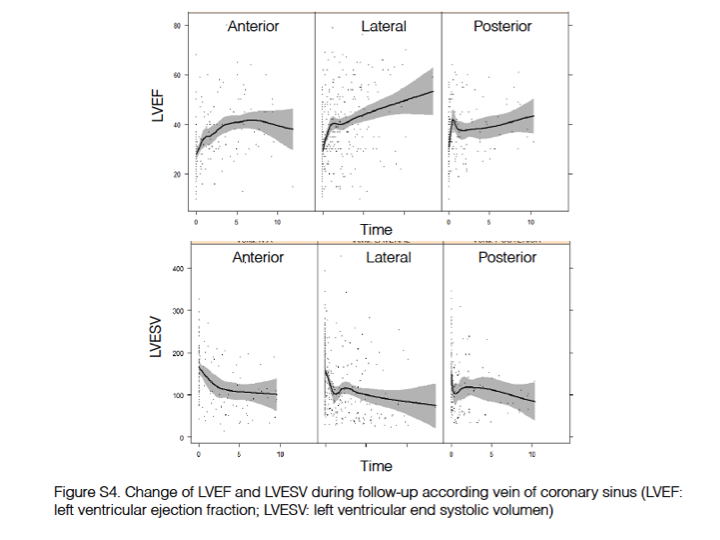

Supplement: Supplementary file 4 — Fig S4 [file JOA3-37-653-s005.tiff]

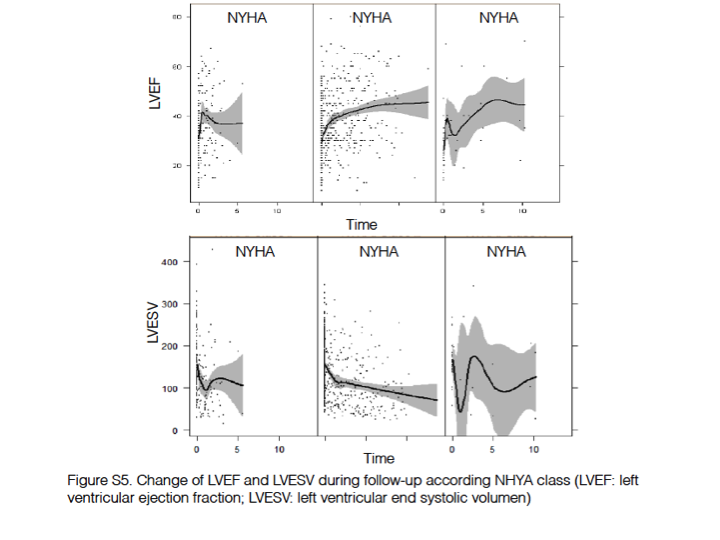

Supplement: Supplementary file 5 — Fig S5 [file JOA3-37-653-s002.tiff]
